# Integrating human rights approaches into public health practices and policies to address health needs amongst Rohingya refugees in Bangladesh: a systematic review and meta-ethnographic analysis

**DOI:** 10.1186/s13690-018-0305-1

**Published:** 2018-10-11

**Authors:** Nidhi Wali, Wen Chen, Lal B. Rawal, A. S. M. Amanullah, Andre M. N. Renzaho

**Affiliations:** 1 Humanitarian and Development Research Initiative, School of Social Sciences and Psychology, Western Sydney University, Locked Bag 1797, Penrith, New South Wales 2751 Australia; 2 School of Public Health at the Sun Yat-sen University, 74, Zhongshan Road II, Guangzhou, 510080 People’s Republic of China; 3 International Centre for Diarrhoeal Disease Research, 68, Shaheed Tajuddin Ahmed Sarani, Mohakhali, Dhaka, 1212 Bangladesh; 4 Department of Sociology at the University of Dhaka, Dhaka, 1000 Bangladesh; 5 School of Social Sciences and Psychology, Western Sydney University, Penrith, Australia

**Keywords:** Bangladesh, Health, Human rights, Rohingya, Refugees, Refugee camps, Statelessness

## Abstract

**Background:**

The Rohingya people of Myanmar are one of the most persecuted communities in the world and are forced to flee their home to escape conflict and persecution. Bangladesh receives the majority of the Rohingya refugees. On arrival they experience a number of human rights issues and the extent to which human rights approaches are used to inform public health programs is not well documented. The aim of this systematic review was to document human rights- human rights-related health issues and to develop a conceptual human rights framework to inform current policy practice and programming in relation to the needs of Rohingya refugees in Bangladesh.

**Methods:**

This systematic review was conducted using the 2015 Preferred Reporting Items for Systematic reviews and Meta-Analysis guidelines. Eight computerized databases were searched: Academic Search complete, Embase, CINAHL, JStor, Pubmed, Scopus, SocIndex, and Proquest Central along with grey literature and Google Scholar. Of a total of 752 articles retrieved from the eight databases and 17 studies from grey literature, 31 studies met our inclusion criteria.

**Results:**

Using meta-ethnographic synthesis, we developed a model that helps understand the linkages of various human rights and human rights-related health issues of Rohingya refugees. The model highlights how insufficient structural factors, poor living conditions, restricted mobility, and lack of working rights for extended periods of time collectively contribute to poor health outcomes of Rohingya refugees.

**Conclusion:**

This review provides a human-rights approach to frame actions both at program and policy level in a sustained way to address the health needs of Rohingya refugees in Bangladesh. Such policy actions will focus on finding long term solutions for integrating the Rohingya population while addressing their immediate rights issue.

**Trial registration:**

This systematic review has not been registered.

## Introduction

The global refugee crisis has led to a sharp increase in the number of forcibly displaced population from 59.5 million in 2014 to 65.6 million in 2016 [1, 2]. Additionally, United Nations High Commissioner for Refugees (UNHCR) estimates that at least 10 million people were stateless or at a risk of statelessness in 2016 [2]. While the term “refugee” is used to describe any person unable or unwilling to return to their home country due to a well-founded fear of being persecuted [3], a stateless person is someone who is not considered as a national by any state under the operation of its law [4]. Accessing basic rights such as healthcare, employment, education and freedom of movement is often impossible for stateless people. Lack, denial or loss of nationality underlies the exclusion of affected individuals from membership in the community, to the point of instigating discrimination and oppression in certain cases. [2, 4]

The Rohingya people of Myanmar are one of the largest groups of stateless refugees in the world [5] accounting to one in seven of the global population of stateless people [6]. Majority of the Rohingya people are not considered to be citizens by the Myanmar Government, which argues that Rohingya people are originally from Bangladesh [7, 8]. In order to avoid conflict and persecution, Rohingya refugees have been fleeing Myanmar in large numbers to nearby countries, primarily Bangladesh, Malaysia and Thailand. Bangladesh has been the preferred destination for Rohingya refugees due to the close proximity and matching religion [9]. Since 1948, Bangladesh has hosted a majority of Rohingya refugees as they came to Bangladesh in three major influxes in 1977–78, 1992 and 2016–17 [10, 11]. Initially the Government of Bangladesh (GoB) positively received the Rohingya refugees and provided adequate support including relief, temporary shelters, food, medical care, and sanitation. However, after 1992 influx of over 250,000 refugees, the GoB attempted a large scale repatriation of Rohingya refugees back to Myanmar. Since this repatriation was not entirely voluntary, many of the repatriate Rohingya refugees returned back to Bangladesh within a decade post repatriation [5, 12].

Consequently, Rohingya refugees entering Bangladesh after 1992 were not officially recognized as refugees by the GoB and despite the repeated influx of Rohingya refugees entering Bangladesh only around 33,000 Rohingya are recognised as official registered refugees and reside in two official UNHCR-led official camps in Cox Bazaar district [13]. While more than 200,000 unregistered refugees living in unofficial makeshift camps [13]. Additionally, recent increase in violence in Myanmar has caused large numbers of Rohingya refugees to cross the border to Bangladesh, making the total number of new arrivals to 620,000 in November 2017 [11], most of whom are undocumented refugees.

With more than twenty years of continuous camp settlements, the current Rohingya refugee situation in Bangladesh has become one of the most protracted in the world [9]. Bangladesh is not a signatory to the 1951 Refugee Convention or its 1967 Protocol and neither is it party to the 1954 and 1961 Convention on Statelessness [5]. The poor socioeconomic condition in Bangladesh with poverty, over population and susceptibility to natural disasters and climate change further complicates finding a durable solution for the Rohingya refugees in the region [14, 15]. The focus of program and policy has been to provide short term relief assistance with a lack of emphasis on finding long term solutions to ensure protection and integration of Rohingya refugees. While UNHCR and other humanitarian actors are able to access and assist only 10% of the estimated Rohingya refugee population, those residing in the makeshift settlements or living as undocumented refugees live in emergency-like conditions and have been identified as ‘persons of concern’ by the UNHCR [5]. Whether living in makeshift settlement or registered camps or in local community areas, the Rohingya refugees have been deprived of their basic human rights of healthcare, employment, education and freedom of movement. They have been subject to miserable living conditions marked by exposure to violence, local hostility, and various forms of discrimination [9]. These conditions have also important public health implications for the Rohingya refugees, where the World Health Organisation (WHO) has graded the present health situation in Cox Bazaar at level three i.e. the highest possible emergency condition [16].

Despite these challenges, there is limited understanding of the complex interplay of human rights issues and health outcomes and a lack of an appropriate human rights framework to inform public health interventions. This study aims to comprehensively document and review human rights-related health issues of Rohingya refugees living in Bangladesh. Thus it attempts to develop a human rights framework that can serve as a useful tool for program and policy for improved health outcomes.

## Methods

### Search strategy

For the purpose of this study, health is defined as the overall well-being [17]. Article 23 of UNHCR’s 1951 Convention, mandates that refugees are to have guaranteed access to public relief services, including health, on par with host country citizens [18]. Conceptualised according to the 2015 Preferred Reporting Items for Systematic reviews and Meta-Analysis guidelines [19], this systematic review considered both peer reviewed and grey literature [20, 21]; and included a combination of mixed methods, qualitative and quantitative. A list of relevant text words and/or corresponding controlled vocabulary according to each database was generated and used to comprehensively search eight computerized bibliographic databases: Academic Search complete, Embase, CINAHL, JStor, Medline, Scopus, SocIndex, and Proquest Central. The following combination of subject headings and keywords were used:

Equal right* [MeSH/Subject Heading] OR Health OR Human right OR Human right violation* OR exploitation of human* OR human trafficking.

AND

Refugee*[MeSH/Subject Heading] OR Rohingya women OR Rohingya refugee OR Burmese refugee* OR Rohingya Muslim.

AND

Bangladesh* [MeSH/Subject Heading] OR Bangladesh region* OR Bangladesh refugee camp*.

Additionally, key words were used for searching grey literature in key organisations websites, including of Ain o Salish Kendra (ASK)- A Legal aid and Human Rights Organisation, Amnesty International, Asian Human Rights Commission, Bangladeshi Red Crescent Society- International Committee of the Red Cross (ICRC), Food and Agriculture Organisation (FAO), Médecins Sans Frontières (MSF), Human Rights Watch, International Organisation of Migration (IOM), Internal Displacement Monitoring Centre, United Nations organisations including United Nations High Commissioner for Refugees (UNHCR), and United Nations Children’s Fund (UNICEF), and World Food Program [22]. Google scholar was searched to include any missed articles and reports. Reference lists of all documents were further scanned for any relevant articles and reports.

### Inclusion and exclusion criteria

Included in this paper were peer-reviewed papers, reports, working papers, and theses or dissertations published in English between 1960 to July 2017; that focused on health and human rights issues of Rohingya refugees in Bangladesh. Editorials, opinion pieces, books and book reviews and papers published in a language other than English were not included. As the scope of this research is limited to the Rohingya refugees living in Bangladesh, reports and articles outside this scope were equally excluded. The study did not attempt a multi country review that includes the other receiving countries such as Malaysia and Thailand due to dissimilarities in states’ policies which have varied in different time frames [23]. We set 1960 as the baseline year because after the military coup in Myanmar in 1962 the Rohingya refugees started migrating in Bangladesh to escape persecution and human rights violations. They were forced to leave Myanmar, then Burma, to seek security in neighbouring nations of Bangladesh, Malaysia and Thailand [24, 25].

### Study selection process

**Fig. 1:**
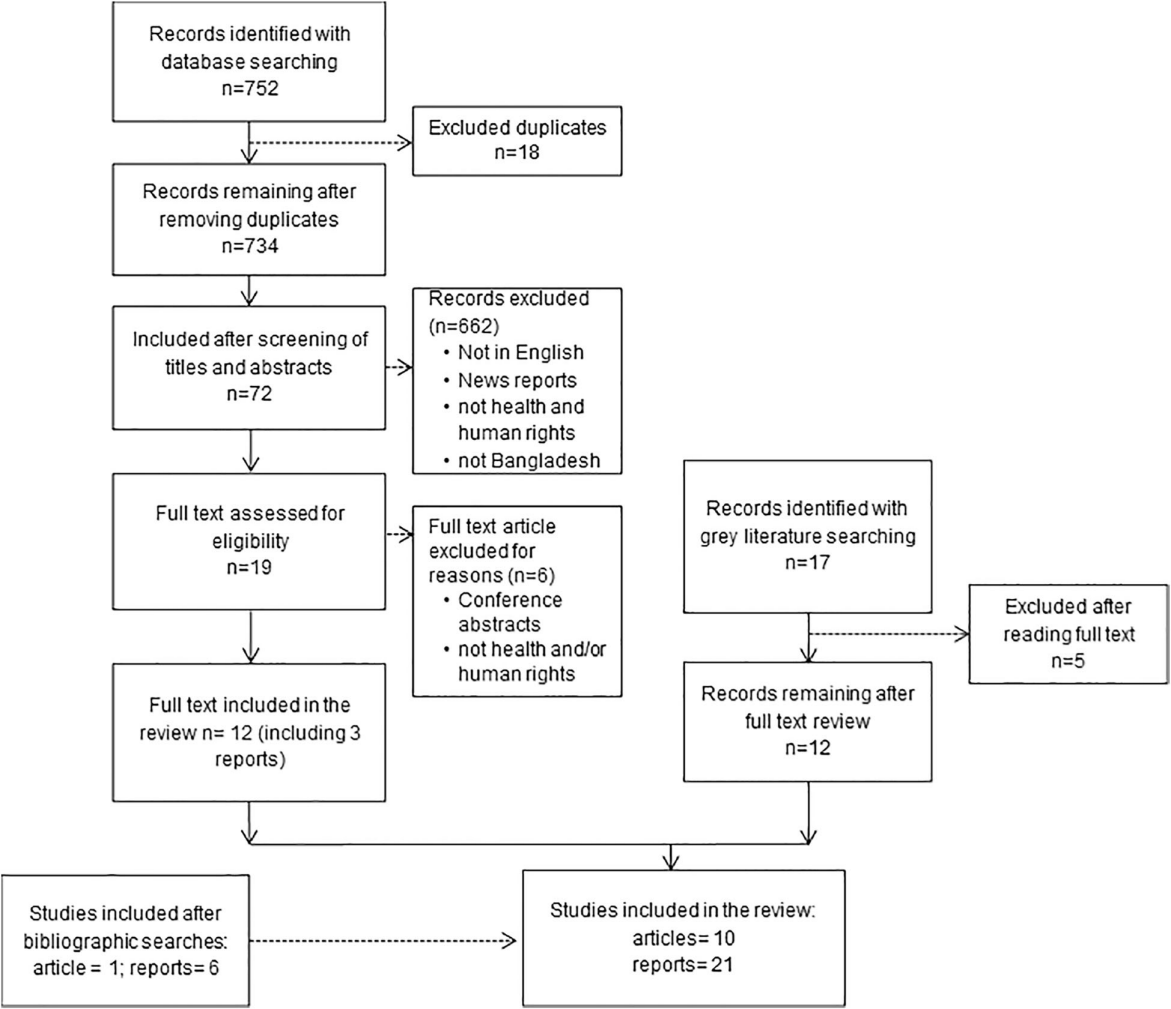
Study selection process

The researchers followed a three staged screening approach to examine the studies eligibility for inclusion. Studies were screened by title to eliminate any duplicates followed by screening of titles to remove any obviously irrelevant studies followed by screening of abstracts to confirm eligibility and relevance. Study selection process is summarised in Fig. 1. A total of 752 articles were retrieved from eight databases. After removal of duplicates, 734 articles were retained. A screening of titles resulted in exclusion of 662 articles. The abstract of the remaining 72 articles were read and screened which led to exclusion of 53 articles. The full texts for the remaining 19 articles were reviewed: seven articles were further excluded and 12 articles were deemed eligible for final inclusion of which nine were peer reviewed articles and three were reports. Additionally, grey literature search including screening of organisation websites and google scholar provided another 17 reports. Full text screening of the reports led to the exclusion of five reports. A manual search of the bibliographic references of all the retained articles and reports identified an additional six reports and one article, thereby a total of 10 articles and 21 reports were included for final review.

The final results were compared to ensure that a consistent approach was taken to evaluating the literature based selection criteria. In cases of discrepancy, consensus was agreed through discussion by two researchers (NW and WC) and where necessary, reviewed by the third researcher (AR).

### Data extraction and synthesis

Two researchers (NW and WC) independently extracted data into their endnote libraries. Data extraction was done using a piloted form. The data extracted included: study details (such as author’s name, year of publication, study design, intervention), study aims and objectives, study characteristics (including sample setting, population). Grey literature was extracted using similar details, by the primary researcher (NW) and subsequently reviewed by two researchers (NW and WC).

### Quality assessment

Two researchers (NW and WC) independently assessed the quality of included studies to minimise errors while maintaining consistency [26]. The methodological quality for qualitative studies was evaluated using the Critical Appraisal Skills Programme (CASP) criteria tool [27]. The CASP tool is commonly used for quality appraisal of qualitative studies [28–30]. The included qualitative studies were rated on a ten point criteria, including study aims, methodology, design, recruitment strategy, data collection, reflexivity, ethical issues, data analysis rigour, clear statement of findings and research value. The Strengthening the reporting of observational studies in Epidemiology (STROBE) checklist was used as a guide to assess the quality of the quantitative studies reviewed [31]. The checklist consists of 22 items, after the initial assessment of all reviewed studies based on the 22 STROBE items, the items were further collapsed into 7 quality-appraisal criteria: sample size, sampling methodology, responses rate, outcome measures, statistical analyses, study limitation and ethical consideration. Mixed methods studies were assessed based on the MMAT (mixed methods appraisal tool) by Pluye and colleagues [32], using a three point criteria of objective, data collection and results. Although MMAT is new and under development it has substantive theoretical validity, is content validated and has been tested for efficiency and reliability [33, 34]. Grey literature was appraised with the AACODS tool that looks at authority, accuracy, coverage, objectivity, date and significance [35]. This tool is being widely recognised by academicians and researchers for appraisal of grey literature. The primary researcher (NW) read the full text of eligible studies and rated each study based on the quality criteria. The secondary researcher (WC) rated a random sample of 13 studies of 31 studies. The scores given by the two researchers were compared and any concerns and discrepancies were resolved with discussion amongst the two researchers (NW and WC) and unresolved discrepancies were independently reviewed by the third researcher (AR).

### Analysis

Due to the heterogeneity of the included quantitative studies in terms of design, settings, and objectives, a meta-ethnographic approach was adopted to synthesise the qualitative data, which was complemented by a descriptive narration of findings for the quantitative studies [36]. The meta-ethnographic approach allowed the analysis to develop a line of research argument synthesis by systematic translation and comparison between studies. The line-of-argument syntheses enables to create new models, theories, or understanding rather than a description of the synthesised papers [36]. All studies were included in the synthesis, where findings from the qualitative studies were juxtaposed with those of quantitative studies as part of the triangulation process. The meta-ethnographic approach involved four stages:

### Identifying metaphors and themes

The studies were read and re-read to gain familiarity with the data and identify themes and patterns in each study. The data were extracted from each of the study verbatim to ensure not to lose any important data. This was validated by revisiting the aims of the study. This process further facilitated in identifying the themes and sub-themes for each study, which were usually found in the results section of the studies.

### Determining how the studies are related

The thematic analysis for all studies was compared to determine how they are related. Even though they were a large number of studies (*n* = 31) the findings of studies had commonalities that contributed in identifying common categories of how the studies are related. For example, structural factors, political and economic factors, social factors, health and well-being and so on.

### Reciprocal translation of studies

We compared the themes and sub-themes of one study to that of another study and so on across all studies. Translation entails comparison and matching of themes across papers to ensure that the key themes across studies are captured. This also ensured to reduce and streamline the themes while identifying them with each of the categories as mentioned in the above step. The primary researcher (NW) undertook these steps with regular consultation with the other authors (WC and AR).

### Synthesizing translations

**Fig. 2:**
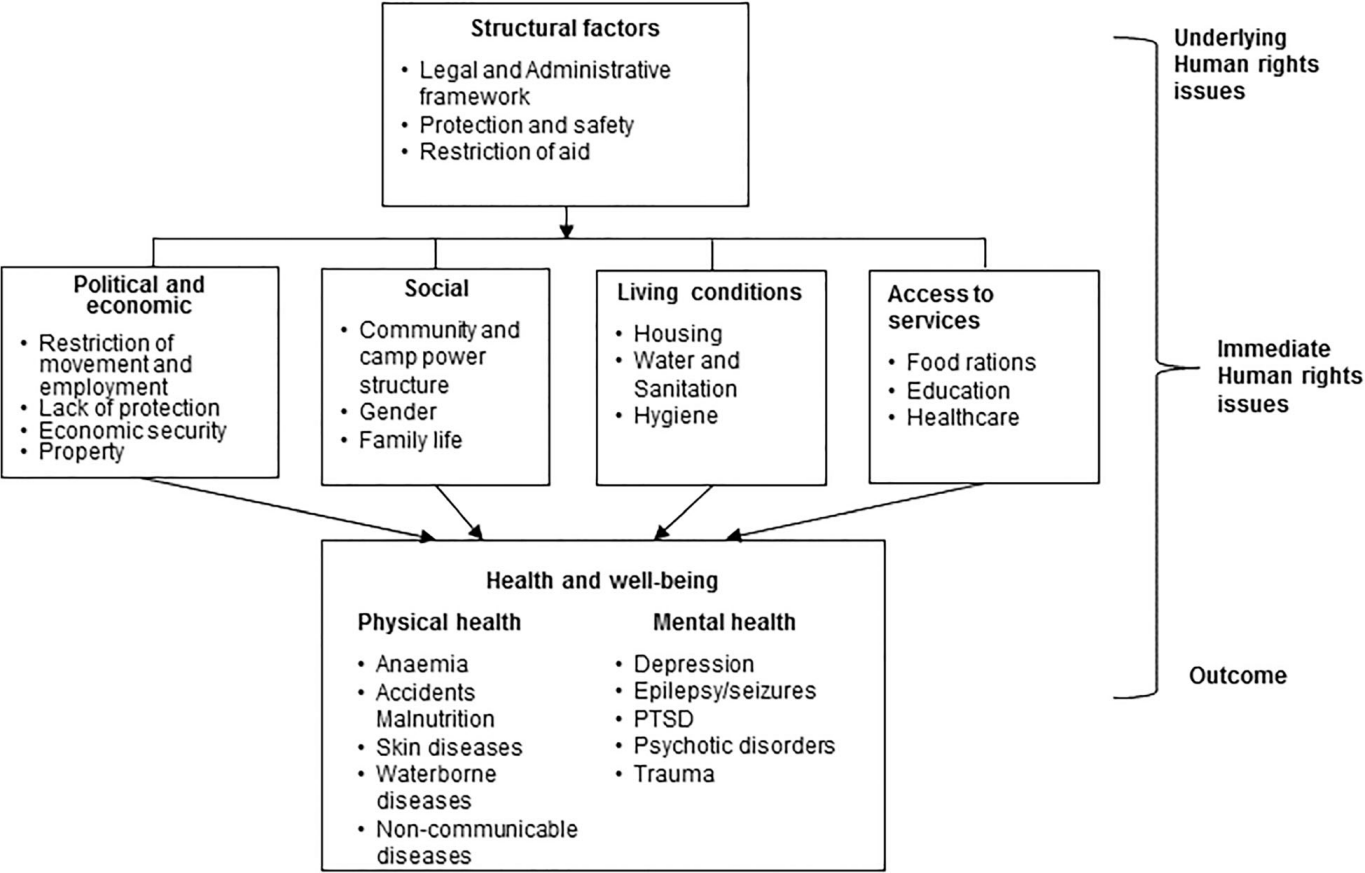
Description of findings

The process of translation between studies was followed by new interpretation of data and developing a line of argument. The team formed the line of argument and produced a model (Fig. 2) with the description of findings.

## Results

A total of 10 articles and 21 reports were included for the final review, as shown in Fig. 1. Most of the studies included were qualitative. However, studies identifying health issues of Rohingya refugees were predominantly quantitative and contributed positively to the overall findings. It is useful to highlight that major studies in the review are those conducted in the official refugee camps. The camps referred to in the findings are these official camps and additional findings from unofficial or makeshift camps are clearly identified otherwise see Tables 1 and 2.

**Table 1:** Characteristics of peer reviewed studies

Study	Title	Aims	Study design	Sample	Intervention	Quality appraisal*
1. Akhter S, Kusakabe K. (2014) [24]	Gender-based Violence among Documented Rohingya Refugees in Bangladesh	Highlights the gender-based violence among documented Rohingya refugees living in the Kutupalong camp in Bangladesh.	Qualitative, Direct interviews	*N* = 24 females and 19 males	No	7/10 High quality
2. Crabtree, K (2010) [45]	Economic Challenges and Coping Mechanisms in Protracted Displacement: A Case Study of the Rohingya Refugees in Bangladesh.	Explore the desires and concerns of refugee populations surviving without adequate aid in order to explore risks associated with income-generating activities and the possibilities for livelihood support.	Qualitative, Interviews and focus group discussions	*N* = 127	No	8/10 High quality
3. Khan, M.U. & Munshi, M.H. (1983) [59]	Clinical illnesses and causes of death in a burmese refugee camp in Bangladesh	To identify clinical illnesses and causes of death amongst Burmese refugees in Leda camp in Bangladesh.	Quantitative	*N* = 954	No	3/7 Medium quality
4. Milton, A. H. et al. (2017) [9]	Trapped in statelessness: Rohingya refugees in Bangladesh	Highlight the Rohingya refugee crisis in Bangladesh, with special emphasis on their living conditions.	Qualitative, literature review and interviews	*N* = 20 Rohingya refugees and other stakeholders	No	8/10 High quality
5. Mahmood SS et al. (2016) [6]	The Rohingya people of Myanmar: health, human rights, and identity	Outlines the historical events preceding this complex emergency in health and human rights.	Qualitative	NA	No	6/10 Medium quality
6. Prodip Alam, M. (2017) [54]	Health and Educational Status of Rohingya Refugee Children in Bangladesh	Explores the educational and health status of Rohingya refugee children with specific attention to gender issues.	Qualitative, key informant interviews	*N* = 16 and other stakeholders	No	9/10 High quality
7. Riley, A. et al. (2017) [53]	Daily stressors, trauma exposure, and mental health among stateless Rohingya refugees in Bangladesh	Examined trauma history, daily environmental stressors, and mental health outcomes for Rohingya adults residing in Kutupalong and Nayapara refugee camps in Bangladesh.	Quantitative	*N* = 148	No	7/7 High quality
8. Tanabe, M. et al. (2017) [56]	Family planning in refugee settings: findings and actions from a multi-country study	A multi country assessment to document knowledge of family planning, beliefs and practices of refugees, and the state of service provision.	Mixed-methods	Survey = 507 households; Facility assessments = 4; DIs =4; FGD participants = 30	No	2/3 Medium quality
9. Ullah AA. (2011) [46]	Rohingya Refugees to Bangladesh: Historical Exclusions and Contemporary Marginalization	Tries to understand the dynamics and severity of reported humiliation by the government of Rohingya population and their marginalization in two camps in Bangladesh.	Mixed-methods	*N* = 134	No	2/3 Medium quality
10. Wijnroks, M. et al. (1993) [58]	Surveillance of the Health and Nutritional Status of Rohingya Refugees in Bangladesh	To determine the health and nutritional status of Rohingya refugees in Bangladesh.	Quantitative	*N* = 161,000	No	5/7 Mediumquality

*Quality appraisal scores: (i) Qualitative: 0–3 poor quality, 4–6 medium quality, 7–10 high quality; (ii) Quantitative: 0–2 poor quality, 3–5 medium quality, 6–7 high quality; (iii) Mixed methods: 0 poor quality, 1–2 medium quality, 3 high quality

**Table 2:** Characteristics of reports

Study	Title	Aims	Study design	Sample	Intervention	Quality appraisal*
1. Access Health International, RTM International	Healthcare at Rohingya Refugee camp: A case study on RTM Initiative	Case study of RTM initiatives of working with Rohingya refugees	Case study	NA	Yes	5/6 High quality
2. Amnesty International 2016 [43]	“We are at breaking point”, Rohingya: persecuted in Myanmar and neglected in Bangladesh	Fact finding research for status and human rights violations of Rohingyas in Myanmar and Bangladesh	Qualitative, Direct interviews & observations	*N* = 55	No	5/6 High quality
3. American International School, Dhaka, 2005 [68]	The Rohingya Refugee situation in Bangladesh	Not stated	Review report	NA	No	2/6 Poor quality
4. Danish Immigration Service, 2011 [48]	Rohingya refugees in Bangladesh and Thailand	Fact finding mission related to situation of RR with regards to asylum claims made in Denmark	Qualitative, Direct interviews	Multiple stakeholders	No	6/6 High quality
5. Forum Asia, 2003 [47]	“We are like a soccer ball, kicked by Burma, kicked by Bangladesh!”: Rohingya refugees in Bangladesh are facing a new drive of involuntary repatriation	To highlight the forced repatriation of RRs	Reports of testimonies	*N* = 57	No	4/6 Medium quality
6. Gawher Nayeem, H. (1994) [49]	Women Refugees in Bangladesh.	Reporting of Oxfam activities in Bangladesh.	Qualitative report	Rohingya refugees	NA	3/6 Medium quality
7. KNOMAD, 2016 [52]	Refugees’ Right to Work and Access to Labour Markets – An Assessment	NA	NA	NA	No	2/6 Poor quality
8. Larkin, Emma & Dunlop, Nic. (2007) [51]	Burma’s forgotten refugees	Status of Rohingya refugees and refugee camps	Report	NA	NA	4/6 Medium quality
9. MSF – Doctors without borders, 2002 [42]	Ten years of Rohingya Refugees in Bangladesh: past, present, future,	Provides an understanding of the condition of the Rohingya refugee now and over the last decade.	Qualitative, Survey and observations	*N* = 118	No	6/6 High quality
10. MSF – Doctors without borders, 2007 [50]	Tal makeshift camp: No one should have to live like this. The Rohingya people from Myanmar seeking refuge in Bangladesh	To document RR’s living condition in a makeshift camp in Bangladesh and its impact on their physical and mental health	NA	NA	No	5/6 High quality
11. Physicians for Human Rights, 2010 [39]	Stateless and Starving: Persecuted Rohingya flee Burma and starve in Bangladesh	NA	Qualitative, consultations and DIs	*N* = 100 households, 25 RRs and 30 other Key informants	No	4/6 Medium quality
12. Refugees Studies Centre, Oxford University, 2001 [55]	Rohingya Refugee Children in Cox’s Bazar, Bangladesh	Aims to provide a background to the Rohingya situation. It then examines the mezo level impacts of displacement on the refugee family and community and its impact on children.	Assessments from secondary data sources	NA	No	6/6 High quality
13. The Arakan Project, 2010 [41]	Unregistered Rohingya refugees in Bangladesh: Crackdown, forced displacement and hunger	Not stated	Qualitative, Direct interviews and field observations	N = 5	No	3/6 Medium quality
14. The Equal Rights Trust, 2012 [40]	Burning Homes and Sinking Lives: A situation report on violence against stateless Rohingya in Myanmar and their refoulement in Bangladesh	Situational analysis	Qualitative, Observations	NA	No	3/6 Medium quality
15. UK Aid and International Organisation of Migration (International Organization of Migration (IOM)), 2017 [61]	Working for a displaced community	Overview of intervention provided by IOM, Bangladesh	NA	NA	Yes	NA
16. UNHCR, University of New South Wales Centre for Refugee Research and the Victorian Foundation for Survivors of Torture, 2007 [44]	Refugee Consultations in Bangladesh	To field test a community based and individual assessment methodology for the early identification of those persons most at risk and traumatised in a refugee community, particularly women and girls, to improve protection, prevention, responses and solutions	Qualitative, consultations and DIs	*N* = 120	No	6/6 High quality
17. UNHCR, 2007 [38]	Bangladesh: Analysis of Gaps in the Protection of Rohingya Refugees	Examines the situation of refugee camp in Bangladesh	Assessments from secondary data sources	NA	No	5/6 High quality
18. UNHCR, 2011 [5]	States of denial	Review UN’s progress in addressing a of protracted refugee situations in Bangladesh	Situation review report	Multiple stakeholders including RR	Yes	5/6 High quality
19. UNHCR and Women’s Refugee Commission, 2012 [57]	Baseline Study: Documenting Knowledge, Attitudes and Behaviours of Rohingya Refugees and the Status of Family Planning Services in UNHCR’s Operation in Cox’s Bazar, Bangladesh	Aims to document the knowledge, beliefs, perceptions and practices of refugees, as well as the quality of services provided in order to improve programming and subsequently increase uptake of FP services among refugee population.	Mixed methods	Survey = 525 households; Facility assessments = 2; DIs =4; FGDs = 6;	No	6/6 High quality
20. UNHCR and World Food Programme, 2012 [22]	The Contribution of Food Assistance to Durable Solutions in Protracted Refugee Situations; its impact and role in Bangladesh: A Mixed Method Impact Evaluation	To assess the role and contribution of food assistance to self-reliance and durable solutions of the affected refugee populations	Mixed methods	*N* = 1069 households;	No	6/6 High quality
21. US dept. of State (2015) [37]	Bangladesh Human Rights Report	To highlight the human rights status in Bangladesh and that of Rohingya refugees in Bangladesh.	Report	NA	NA	6/6 High quality

*Quality appraisal score: 0–1 poor quality, 2–4 medium quality, 5–6 high quality

Findings indicate that a combination of underlying and immediate human rights issues at macro and meso levels respectively interacted to negatively affect the health of Rohingya refugees in Bangladesh. Human rights-related health issues were identified at three different levels: underlying human rights issues (societal level), immediate human rights issues, (household/community level), and health outcomes (individual level).

### Underlying human rights issues: Societal level

The underlying human rights issues were mainly structural factors, including legal and administrative barriers, issues related to protection and safety, and restriction of aid. Indeed, Bangladesh is not a signatory of the Convention and Protocol relating to the Status of Refugees, 1951 [5], and has no legal obligation to protect or safeguard the refugees and asylum within the country. The absence of a national refugee and asylum seekers legal and administrative framework means that Rohingya refugees are exposed to serious protection risks with limited opportunities.

Our findings suggest that Rohingya refugees experienced violence and abuse perpetrated at various levels, within the official camps and outside the camps [5, 37–41]. Violence and abuse have been perpetrated by

- The camp administration, police, and refugee block leaders, *mahjee*: as illustrated by a female refugee, *‘I have reported [my problem] five times to the UNHCR. In my eyes, the UNHCR and the mahjee [block leader] and the police are the same’,* [42]*.*
- Employers and local community outside the camp, as a female refugee summed it up: *‘He asked me to bring tea to his bedroom. I felt very uncomfortable but again I had no choice. So I prepared the tea and went to the bedroom. The owner then suddenly locked the room … and I tried to run away, but he grabbed me hard. At first I tried to shout and fight, but then I realised that I would lose my job. So I gave up the fight and reluctantly let him do what he wanted. I was not able to share this story with anyone because I would not only lose my job, but also be socially stigmatised.’* [24].

Additionally, the camps witnessed increased female-headed households due to abandonment by husbands and family separation due to displacement increasing their vulnerabilities [43, 44]. A single mother of eight noted: ‘*When my husband passed away, everything turned dark. My main concern was about my children. The limited amount of ration was not sufficient for my family’s survival. I started searching for work. Being a woman in a new land and environment, it was very challenging in every aspect’* [24].

Our findings suggest that the GoB did not wish to improve the living conditions and provide safety to the refugees. In 2016, the GoB put restrictions on the aid distributed to newly arrived refugees because it argued that aid distribution would lead to an increase in the influx of newly-arrived refugees [43, 45]. It noted: “*Distribution of relief among the refugees will encourage more Rohingyas to enter the country”* [43]. Another important structural factor that violated the rights of Rohingya refugees is forced repatriation. For example, in 1992 and 1998 the GoB planned a repatriation drive for the return of refugees to Myanmar. The repatriation was not voluntary and beatings and other physical abuse by camp administration and GoB were common to persuade refugees to voluntarily depart [5, 6, 38, 40–42, 46–50]. According to a MSF survey, 63% of refugees repatriated during the 1990s under the voluntary repatriation drive by the GoB did not want to return to Myanmar [42]. Involuntary repatriation also caused families to disintegrate as their family members were forced to leave, as highlighted by a male refugee, ‘*We were with nine in my family. Six were repatriated by force by the camp police. They took my wife, our two children, my brother, father, and mother. My two brothers and I were somewhere else in the camp when our family was taken’* [42].

### Immediate human rights issues: Household/community level

#### Political and economic

Over the many years spent in camps, Rohingya refugees have had restricted mobility and in some cases have not been allowed to go outside the camps without an official permit, primarily to meet a family member in another camp or for medical reasons. These restrictions severely affected their basic rights of mobility, access to livelihoods, food, water, sanitation, and education. Refugees were not allowed to work outside the camps, but with very limited opportunities within the camps, economic constraints and limited food rations many were forced to seek employment illegally outside the camp. This further exposed them to serious risks including: the need to bribe camp authorities to go out as they are prohibited otherwise; harassment by the local police who often targeted them as outsiders and arrested them for working; discrimination by local employers by paying them lower wages and also by the local people who accused the Rohingya refugees for taking away their jobs. These restrictions also forced some refugees to live outside official camps for better livelihood opportunities further compromising their safety [6, 24, 37, 42–46, 48, 50–52].

Lack of money and income-generating activities were of major concern to the refugees. As a male refugee shared his uncertainties about finding work, *‘We are not sure we will get work in the local community. We may get today, but we are not sure about tomorrow, whether the employer will allow me to work there again. I feel uncertain every morning whether I will get any work to do today or not. I cannot even claim the same wage as local people because they [the employers] know that we are illegal, so we have to accept whatever amount they give us*’ [24]. With the current circumstances employers are not punished for refusing to pay wages, paying extremely low wages, or forcing employees to work under unsafe conditions [45].

It is useful to note that the restricted mobility of Rohingya refugee men forced more Rohingya refugee women to look out for work outside the camps. These women’s wages were lower than those of men, while refugee men earned much less than local men. The changing mobility profile along with the increased economic role of women led to more domestic violence and abuse from the refugee community and outsiders[24, 45].

#### Social milieu

Our findings suggest that overcrowding, boredom with no work to engage in over the years along with restricted mobility led to increased inter-familial tensions and a loss of community spirit [48, 51]. The camp community structure was identified as hostile and unsafe with increased power with the authorities and the camp block leader, *mahjee* [24, 42, 44, 49]. As stated by teenage refugee, ‘*The mahjee has destroyed the environment of the camp’* [42]. Although the UNHCR and the GOB have made attempts to replace the *mahjee* system with block committees [38], research highlights that the *mahjees* continues to dominate the camp social structure and the refugees [24]. A female refugee shared her experience, ‘*Life is very hard for me. I do not have any other means to support myself and my family. To avoid going to jail, I have to regularly please the local policemen. I have to share my meagre income and also give them sexual services. I also have to share a percentage of my income with the mahjee to keep my registration at the camp. Sometimes he also forces me to have sex with him by threatening to cancel my registration and send me and my family back to Myanmar. So I have no choice but to do whatever he wants’* [24].

The loss of trust and respect for the institutions and structures that were meant to protect the refugees further led to strained relationships within the camp as a whole, as well as traditional respect and obedience structures within the family. A research highlighted low social functioning and satisfaction amongst Rohingya refugees and slight satisfaction with personal relationships [53]. The extended camp life with its restrictions and embedded gendered norms within the Rohingya culture impacted relationships and family harmony within families with increased alcoholism and domestic violence. As pointed by a female refugee about her husband, *‘He is very frustrated with life and society. Even though he was a very nice man before, society has changed him. He is not nice anymore. He passes his time by drinking. If I tell him to stop drinking, he starts to physically and verbally abuse me’,* [24].

Family separation: The experience of displacement led to increased family separation within Rohingya refugee communities, as many families had lost their family members during persecutions in Myanmar or those who were seriously injured to cross the border into Bangladesh. Forced and involuntary repatriation movement by GoB also separated many families [5, 42, 44, 48].

Gender: Gender-related issues are common within the Rohingya culture and have persisted within the camps over the years. Our findings reveal that limitations of refugee life within the camps further intensify these existing gender distinctions and discriminations. In Rohingya culture the sons are viewed income-earners in the family, hence an asset and daughters are considered a burden [54]. Within refugee families while the first girl of a family may be kept longer for her domestic contribution, second and third daughters become superfluous and therefore to be married off as soon as possible, or in some cases even sold to traffickers [24, 49, 54, 55]. As a refugee parent summed it up: ‘*Daughters are good for nothing. When I will be old my son will take care of me. I don’t want to spend more money on my daughters. As a result, I have already let marry two of my daughters before completing their education’* [54].

#### Living conditions

The GoB constructed semi-permanent structures 1992 during the first major wave of Rohingya refugee population taking refuge in the country. In order to not encourage more influx, the GoB did not build permanent structures until 2006 when they allowed repair and maintenance followed by construction of larger and more permanent shelters in accordance with international standards in two official camps. However, the camps continue to be overcrowded accompanied with limited water and poor sanitation and hygiene facilities [5, 9, 51, 54]. A refugee shared his condition, *“(We) wish to highlight our living conditions. It is really crowdy and we cannot move. There is smoke in the sheds. We get a lot of diseases and children get sick. Water supply is totally insufficient. We get it only in the morning and only two buckets and tomorrow it will be the same again”* [44].

There was also concern of the poor gender consideration for the available sanitation facilities, which have been built together in the official camps for male and female. This made it very uncomfortable for females to use these facilities at the same time male used them. There were also experiences of rapes and sexual assaults of Rohingya refugee women during making the trip to these facilities, making them more vulnerable. A teenage girl points out, ‘*The doors are damaged, so people can see inside. I often wait until dark to go to the toilet, but it is dangerous’* [42].

The unofficial camps however continue to have semi-structured shelters with destitute living conditions. For example, in a MSF report [2007] at the Tal makeshift camp, the average family size was 5.1 but up to 12 people lived in one shelter [50]. These unofficial makeshift camps had fewer latrines, below the SPHERE standard of one latrine per 20 individuals. They had poor hygiene and sanitation facilities such as open sewers and substandard sanitation systems. During the rainy season majority of these shelters were flooded. These sanitation practices across the official and unofficial camps have persisted over the years and were an increasing cause of diarrhoea amongst the children and posed a public health threat to the refugees living there [5, 6, 39, 42, 43, 46, 50, 51, 54].

The official and unofficial camps also continued to have shortage of water with limited potable water per person and family [9, 42, 46, 50, 51]. A female refugee in a household of seven shared her experience of dealing with this shortage, *“I have to spare water for my other family members. So sometimes I bathe only two to three times per month”* [42].

#### Access to services of food, education and healthcare

Food security and malnutrition along with lack of sufficient cooking fuel were identified as serious problems in the camps. There were many contributing factors such as inaccurate family books, unregistered new born whom the government failed to register, more members per family to feed than those recorded and sharing of rations with other unregistered refugees. [5, 9, 42–44, 54] As highlighted by a refugee mother*, ‘My 10-year-old son died four months ago from starvation, and now my daughters cry every night for food. I leave home twice a day to beg for food and money’* [39]. Another refugee male shared his dilemma, ‘*I receive rations for five people, but there are 10 people in my house. I borrow food from my neighbours, or I sell or trade other things to get more food’* [42].

There was also poor food quality and diversity in the food provided. A refugee shared his plight, “*We have been eating the same foods for 10 years. Who can eat only rice and [dahl] every day, for 10 years?”,* [42]**.** Although the food meets the international standards of calorific intake it lacks sufficient animal proteins and vegetables which were also identified as probable causes of high incidence of high malnutrition in the camp [22, 38]. A WFP evaluation reports clearly pointed out a low household dietary diversity score (HDDS) of the refugee families [22].

Education is provided with limited facilities and is available only in the two official camps. Formal education for primary and kindergarten was provided since 1997 and in 2007 the GoB established secondary education, with 21 primary schools and 2 secondary schools in the camps. However, there was high drop-out rate due to the intention to earn money by children. Gender distinction in the provision and uptake of education with a preference to males was also identified. But refugees believe that education is the only hope for their future and wanted to provide their children with education, and would often take many risks for the same [5, 9, 38, 44–46, 54].

Healthcare: Basic healthcare services were provided by International organisations in the camps with a provision of referral to government hospitals. However, government authorities did not encourage formal or regular access by Rohingya refugees to public health care system of Bangladesh. [5, 9, 37, 38, 42, 44, 47, 54]. A government doctor clearly points out, ‘*The Rohingyas are exhausting hospital resources. Treatment should be for Bangladeshis. Staff was told not to treat them (Rohingyas) even if patients are dying in front of them’,* [50]. Mother and child health services were given prime importance in the camps; there was also high awareness and uptake of family planning methods and services [56, 57].

### Health outcomes: Individual level outcome

#### Health and wellbeing

Findings suggest emergence of physical and mental health outcomes amongst the Rohingya refugees as a result of underlying and immediate human rights issues. This has exacerbated with extended periods of living in the camp, in some cases of 15–20 years, background of trauma and torture and a persistent uncertainty about the future.

Common physical health conditions identified were: high prevalence acute of malnutrition amongst Rohingya children, where 20% of children suffer from wasting which has although halved from 1992, when wasting was noted in 40% of newly arriving Rohingya children but is still higher than the recommended international standard [6, 58]. Stunting, or low height for age, also known as chronic malnutrition or stunting was prevalent amongst 60% of Rohingya refugee children in Bangladesh, a rate 50% higher than the rest of the Bangladesh population (which itself has high rates of malnutrition) [6, 9]. Latest mortality rates [in 2015] show the infant mortality rate (IMR) in one of the two official camps [Nayapara] was at 54.5% and the neonatal mortality at 12% in the other official camp [9]. Already born with low birthweight, poor nourishment continues throughout the life of infants born into Rohingya refugee families. MSF highlighted that as early marriage and early pregnancy were commonly observed in this community poor nutrition in female child could affect neonatal and infant mortality rates and pose a potential danger to both the mother’s and child’s health [42]. Poor nutrition and anaemia in children were identified as a consequence of food insecurity and lack of food diversity. Communicable diseases such as respiratory tract infections, diarrhoea, skin diseases, measles and water borne diseases have been persistent since the 1990’s given the poor and inadequate hygiene and sanitation facilities [6, 9, 39, 42–44, 46, 50, 54, 56, 58–61]. Milton et al. (2017) also highlight the prevalence of non-communicable diseases and injuries caused due to accidents and self-harm in some cases [9].

Mental health conditions such as chronic anxiety, grief, depression and post traumatic disorders (PTSD) were common. A study (2017) conducted in the Rohingya refugee camps revealed that 36% of its participants had PTSD symptoms while 89% had depression symptoms [53]. Other mental health issues were epilepsy, seizures and psychotic disorders. Findings highlight that daily stressors associated with life in the refugee camps, over extended periods of time played a significant role in the mental health outcomes and were identified of more immediate concern than the past traumatic events [9, 44, 50, 53].

## Discussion

The study findings highlight poor health outcomes of the Rohingya refugees residing in refugee camps in Bangladesh. It identifies human rights issues of prolonged displacement, destitute living conditions, lack of social milieu, limited access to services, and the persistent scarcity of resources as key contributing factors of poor physical and mental health outcomes. Secondly the study determines that these factors are interdependent and work together with underlying policy and rights issues which further increase their vulnerability to access and utilise services. Finally it provides a model that clearly demonstrates these linkages, serving as a rights’ based assessment indicator to explore factors affecting physical and mental health of a population.

The most common health conditions identified in refugee camp settings are those revolving around hygiene, respiratory problems and infectious diseases which are often attributed to the environment conditions [62]. Such findings are similar to results reported in other over crowded refugee camps. For example, evidence from Kenyan refugee camps suggests linkages between living in overcrowded spaces to respiratory illness[63] with similar findings that link environmental factors contributing to health conditions from refugee camps in Senegal, Palestine and Mexico [62, 64]. In addition to the physical limitations within camps there are also mental health consequences of persecution, war, and historical trauma combined with daily environmental stressors associated with prolonged displacement, statelessness and life in refugee camps. There is now growing literature that specifically identifies the increased role of daily environment stressors in mental health outcomes [65–67]. Further the global refugee crisis presents a clear relationship between the refugee problem and the issue of human rights,[18] as also highlighted in the study findings. With the growing understanding of the interdependence of various structural and immediate factors that collectively determine the realisation of human rights of including the right to health. The 1951 convention and 1967 Protocol related to the status of refugees and ‘new refugees’ clearly outlines minimum standards of treatment of refugees, including the basic rights to which they are entitled including the right to employment, access to services including health and education, social security and freedom of movement [18].

However, in the case of Bangladesh, the complex interplay of underlying and immediate human rights of refugees and health outcomes has limited understanding and needs to be addressed through a comprehensive approach. Presently there is a lack of an appropriate human rights framework to inform public health interventions. This review provides a model that helps identify human framework to understand and explore human rights factors affecting physical and mental health of a population. Thereby, it positively contributes to fill the existing knowledge gap in this growing area of research. It highlights the need to apply human-rights approach to frame actions both at program and policy level to ensure improved health outcomes of the Rohingya population. Such policy actions will focus on finding long term solutions for integrating the Rohingya population while addressing their immediate rights issue.

It is worth noting that studies in this systematic review were restricted to research only in English, and only Rohingya refugees residing in Bangladesh, hence limiting the external validity of our findings. The study did not attempt a multi country review to include other countries receiving, such as Malaysia and Thailand. This is due to the dissimilarities in states’ policies which have varied different time frames. Notwithstanding these limitations, findings from this study will enable policy makers to ascertain the factors associated with mental and physical health outcomes of Rohingya refugees.

## Abbreviations

AACODS: Authority, Accuracy, Coverage, Objectivity, Date and Significance
ASK: Ain o Salish Kendra
CASP: Critical Appraisal Skills Programme
FAO: Food and Agriculture Organisation
GoB: Government of Bangladesh
ICRC: International Committee of the Red Cross
IOM: International Organisation of Migration
MMAT: Mixed Methods Appraisal Tool
MSF: Médecins Sans Frontières
STROBE: Strengthening the reporting of observational studies in Epidemiology
UNHCR: United Nations High Commissioner for Refugees
UNICEF: United Nations Children’s Fund
WHO: World Health Organisation
